# Global Transcriptome Analysis Reveals Small RNAs Affecting *Neisseria meningitidis* Bacteremia

**DOI:** 10.1371/journal.pone.0126325

**Published:** 2015-05-07

**Authors:** Luca Fagnocchi, Silvia Bottini, Giacomo Golfieri, Laura Fantappiè, Francesca Ferlicca, Ana Antunes, Serafina Guadagnuolo, Elena Del Tordello, Emilio Siena, Davide Serruto, Vincenzo Scarlato, Alessandro Muzzi, Isabel Delany

**Affiliations:** 1 Novartis Vaccines and Diagnotics, Siena, Italy; 2 Department of Pharmacy and Biotechnology, University of Bologna, Bologna, Italy; University of Würzburg, GERMANY

## Abstract

Most bacterial small RNAs (sRNAs) are post-transcriptional regulators involved in adaptive responses, controlling gene expression by modulating translation or stability of their target mRNAs often in concert with the RNA chaperone Hfq. *Neisseria meningitides*, the leading cause of bacterial meningitis, is able to adapt to different host niches during human infection. However, only a few sRNAs and their functions have been fully described to date. Recently, transcriptional expression profiling of *N*. *meningitides* in human blood *ex vivo* revealed 91 differentially expressed putative sRNAs. Here we expanded this analysis by performing a global transcriptome study after exposure of *N*. *meningitides* to physiologically relevant stress signals (e.g. heat shock, oxidative stress, iron and carbon source limitation). and we identified putative sRNAs that were differentially expressed *in vitro*. A set of 98 putative sRNAs was obtained by analyzing transcriptome data and 8 new sRNAs were validated, both by Northern blot and by primer extension techniques. Deletion of selected sRNAs caused attenuation of *N*. *meningitides* infection in the *in vivo* infant rat model, leading to the identification of the first sRNAs influencing meningococcal bacteremia. Further analysis indicated that one of the sRNAs affecting bacteremia responded to carbon source availability through repression by a GntR-like transcriptional regulator. Both the sRNA and the GntR-like regulator are implicated in the control of gene expression from a common network involved in energy metabolism.

## Introduction

The essential role of small RNA (sRNA) transcripts for the regulation of many biological functions, especially gene expression, is now well-established for many organisms. In pathogenic bacteria, regulatory sRNA transcripts are a heterogeneous group of molecules, which modulate a wide range of physiological processes through different mechanisms (reviewed in [1, 2]). Such molecules include mRNA 5’UTR riboswitches that are regulated through metabolite-binding [3], the recently discovered CRISPR (clustered regularly interspaced short palindromic repeats) RNAs that interfere with bacteriophage infection and plasmid conjugation [4], dual-function sRNA regulators [5], sRNAs that bind proteins and alter their functions [6], and finally the largest and most extensively studied set of sRNAs that act through base-pairing with mRNAs thus modulating their translation and stability. The base-pairing sRNAs comprise both cis-encoded antisense sRNAs, located on the strand of DNA opposite their mRNA targets and having extensive complementarity to these [7] and the trans-acting sRNAs, acting on multiple remote targets with limited complementarity [2]. The general outcome of sRNA-mRNA interaction is degradation of the duplex by RNase E, but gene expression activation by sRNAs has also been reported [8].

The role of multiple sRNAs in pathogenic bacteria has been elucidated. For example, sRNA activity can be related to metabolic changes and the regulation of homeostasis [9], repression of outer membrane protein synthesis [10–14], adaptation and resistance to stress [15, 16], virulence and pathogenesis [16–19] and quorum sensing system regulation [16].

Trans-encoded base-pairing sRNAs in Gram-negative bacteria generally depend on the hexameric RNA chaperone Hfq, a homologue of eukaryotic Sm-like proteins involved in splicing and mRNA decay, that mediates the sRNA-mRNA interaction [20, 21]. There are multiple mechanisms by which Hfq assists sRNA-target base-pairing; e.g. Hfq increases the annealing rates of RNA molecules [22–24], stabilizes cognate sRNA-mRNA duplexes [25], promotes structural remodelling of sRNA and target mRNAs [26], and increases the local concentration of mRNAs and sRNAs [27].

The central role of Hfq in assisting sRNA regulatory circuits involved in fitness and virulence of many bacterial pathogens is highlighted by the pleiotropic phenotypes of Hfq-knockout strains, which present increased sensitivity to host defence mechanisms and attenuation in animal models [28–33]. Post-transcriptional regulation by sRNAs has also been shown to occur by Hfq–independent mechanisms in Gram-positive bacteria such as *Staphylococcus aureus* and *Bacillus subtilis*, despite the presence of Hfq in these organisms [34, 35].

The role of sRNAs and Hfq-mediated regulation in *N*. *meningitidis* has been investigated only in the last decade. *N*. *meningitidis* is an important Gram-negative obligate human pathogen, carried by up to 15% of healthy individuals in the oro-pharyngeal tract and representing a major cause of septicaemia and meningitis worldwide [36, 37]. During its pathogenesis, *N*. *meningitidis* encounters different stresses and environmental changes, crossing the mucosal epithelium, spreading into the blood and reaching the meninges [38].

To date, a few sRNAs have been identified in *N*. *meningitidis* or the closely related pathogen *Neisseria gonorrhoeae* (gonococcus), and they are involved in a number of critical pathogenic processes including regulation of gene expression, natural transformation and antigenic variation [39–41]. A role for Hfq has been inferred for two of these sRNAs, namely NrrF and AniS [30, 42]. NrrF, which was first identified through a bioinformatic approach, is synthesized during iron starvation and is involved in maintaining iron metabolism and homeostasis [30, 43]. AniS, identified in a microarray screening of transcripts differentially-expressed between wild type and Δ*hfq* mutant strains, is induced in anoxia and may be involved in down—regulation of FNR-repressed genes [42]. A third sRNA has been identified in *N*. *meningitidis* strain H44/76 RNA-sequencing, which was named σ^E^sRNA due to its up-regulation in a mutant in which σ^E^ is highly expressed [44]. This sRNA was subsequently discovered to be the tracrRNA of the Type II CRISPR/cas system of meningococcus, which has been shown to limit natural transformation of the bacterium [41]. Recently, RNA thermo-sensors have been identified in the 5-UTRs of three meningococcal genes that are essential for resistance against immune killing [39]. Interestingly, another novel sRNA has been implicated in antigenic variation of the pilus in gonococcus [40].

Recently, high-density array experiments together with a new bioinformatic tool named chipSAD have been employed in order to investigate differential transcriptional expression of *Neisseria meningitidis* in whole human blood [45]. This analysis revealed the presence of 91 differentially expressed putative sRNAs after incubation in human blood, six of which were successfully validated by 5’-3’ RACE, including NrrF [45].

In this study, we sought to identify sRNAs of *N*. *meningitidis* strain MC58 that are regulated in multiple infection-relevant conditions. Using the previously applied tiling array technology [45] we identified 98 putative sRNAs, which were differentially regulated in at least one of the conditions tested. Eight new sRNAs and 3 additional sRNAs, previously identified to be induced in *ex vivo* blood, were successfully validated by Northern blot [45]. We provide data suggesting that one of these, sRNA 1563–1564_F (Bns1 in reference [45]), is in a regulatory network with a GntR-like regulator and is involved in regulation of metabolic processes. Finally, we evaluated deletion mutants of 7 sRNAs in the *in vivo* infant rat model of bacteremia, identifying the first 4 sRNAs important for meningococcal fitness during infection.

## Materials and Methods

### Bacterial strains and culture growth conditions

The *N*. *meningitidis* strains used in this study were the clinical isolates MC58 and 2996 wild type strains and their derived mutants (S1 Table). All strains were routinely cultured and stored as described previously [46]. Liquid cultures of MC58 were grown in 7 ml volume in a 14 ml tube to mid-logarithmic phase (OD_600_ ~ 0.4–0.5) in modified C6 medium [47] with or without supplementation of 1% (w/v) glucose and to both mid-logarithmic and early stationary phase (OD_600_ ~ 1–1.2) in GC medium (GC-based medium (Difco) with Kellogg’s supplement I and 12.5 mM Fe(NO_3_)_3_). After culturing to mid-log in GC medium, MC58 cultures were exposed to short environmental shocks for 5–12 min. The cultures were added to an equal volume of frozen medium to bring the temperature immediately to 4°C and cells were harvested by centrifugation at 3000 rpm for 20 min for RNA extraction. When required, erythromycin or chloramphenicol were added to a final concentration of 5μg/ml. For the microarray experiments, differential expression under the following seven conditions was assessed: 1) carbon source limitation: by comparing mid-logarithmic cultures grown in GC medium and C6 minimal medium, 2) glucose availability: by comparing mid-logarithmic C6 medium cultures with or without supplementation of 1% (w/v) glucose, 3) stationary phase: by comparing GC medium mid-log cultures to stationary phase cultures, 4) Hfq-regulation: by comparing mid-logarithmic GC medium cultures of the wild type strain to its respective Δ*hfq* mutant, 5) iron chelation: mid-log GC cultures exposed for 5 min with or without 250 μM 2,2-dipyridyl (Sigma), 6) oxidative stress: mid-logarithmic GC cultures exposed for 12 min with or without 135 μM H_2_O_2_ (Sigma), and 7) heat shock: mid-logarithmic GC cultures exposed for 10 min to 44°C or 37°C in a water bath.

### RNA extraction, Microarray design and experimental procedure

RNA for microarray experiments was prepared and labelled as previously described [42]. Briefly, RNA was extracted using the RNAeasy Mini-kit (Qiagen) following manufacturer’s instructions. RNA pools were prepared from three independent bacterial cultures. Three independent pools were prepared for each condition and pairwise 2-colour hybridizations were performed with Cy3 or Cy5 labelled cDNA on microarray slides.

The tiling [45, 48] and gene-based [42] microarray designs and the experimental procedure have been described previously. Briefly, the gene-based oligonucleotide microarray was designed with at least 2 ORF-specific 60-mer oligos to cover 2078 predicted ORFs of *N*. *meningitidis* MC58 strain (Array Express Design ID, A-MEXP-1957). For 80 genes it was not possible to design specific probes and the final coverage was 96.2% (2078/2158 genes). For the tiling array, tiling probes of 60 nt, shifted by 10 bp probes, were included in the design for non-coding regions, either intergenic or antisense, and for coding regions of ORFs less than 300 bp in length. For coding sequences of ORFs longer than 300 bp, the probes designed for the gene-based array were included. The final design includes 6,877 probes for coding and 36,869 probes for noncoding portions of the chromosome in addition to control probes specific to the Agilent technology publicly available from the Array Express database (Design ID, A-MTAB-517). Images were acquired by using an Agilent microarray scanner G2505B at 5 mm of resolution and using the extended dynamic range and were analysed with Agilent Feature Extraction 9.5.1 software. Before any other analysis, we computed an average of replicated probe signals [M = log_2_(Cy5/Cy3) and A = log_10_(Cy3xCy5)/2] within each slide.

### Data analysis: the chipSAD method

The differentially transcribed regions were investigated by applying the previously described chipSAD method [45]. Briefly, the method uses a probe position-based approach to detect differentially expressed regions (Signal AreaS or *SAS*) analysing the level of the continuous probe signal (M = log_2_(ratio)) using pairs of contiguous sliding windows through the genomic coordinates. The chipSAD method was applied for each replica of each experiment identifying 21 lists of putative SAS. We then filtered the SAS for those defined as intergenic. A SAS is defined as intergenic if the region does not overlap an ORF or overlaps it for less than 20% of its length in both strands and its length is less than 800 bases. We set up a new methodology to merge the intergenic SAS lists of different experiments, which are differentially expressed in at least one replica, in a unique list. Comparability and the overlap of the lists were influenced by the specificity of the differential expression in each condition and by the probes at the beginning or at the end of different boundary regions, responding in different ways. The “alignment procedure” consists in the detection of the SAS in different lists that could be considered the same, by requiring a minimum overlap of the boundaries of 30%. Then, the boundaries of the putatively corresponding SAS were recalculated by using a weighted average (based on the M value) of their positions.

We then ranked the intergenic SAS calculating a score index, the Shapley value, employing a game theory approach similar to that described previously [49, 50]. We slightly modified the Shapley value calculation, in order to rank each intergenic SAS by its level of differential expression (the M value), the reproducibility of each SAS behaviour in the three replicas and considering in how many conditions each SAS was differentially expressed. We set up a threshold on the obtained Shapley values considering the total variance and the shape of its distribution, obtaining a subset of 592 intergenic SAS that may be the most significant putative intergenic differentially regulated signals.

To generate a curated list of putative sRNAs, results from the microarray experiments were manually inspected with the support of the Artemis genome browser [51]. Small transcripts as annotated in the JCVI CMR database, such as rRNAs, tRNAs, and known repeated regions (ATR, Correia Elements, dSR3, REP REP2, REP3, REP4 and REP5) were eliminated. An in house Perl algorithm was developed to automatically assess the presence and the distance of putative terminators (Rho-independent terminators or other stem loop-forming motifs) within 1000 bases following the 3’ end of differentially transcribed intergenic signals. Rho-independent terminators were predicted using TransTermHP algorithm [52] and putative stem loop-forming motifs were predicted using the palindrome algorithm included in the EMBOSS 5.0.0 software package [53]. Curation for positioning of the signal and co-regulation with the upstream or downstream genes was also considered, which allowed definition of the signals as distinct intergenic sRNAs, 5’- or 3’-UTR sRNAs or overlapping antisense sRNAs. Finally, transcripts longer than 500 nt were excluded. The criteria for manual curation resulted in a list of 98 intergenic SAS which were considered the most representative of putative sRNA candidates (S2 Table).

### Primer extension and Northern Blotting

Primer extension and Northern blotting were performed as reported previously [42]. Briefly, 5 pmol of radioactively-labelled oligos (S1 Table) were incubated with 20 μg of total RNA in the presence of 5U of AMV reverse transcriptase (Invitrogen) for 1 hour at 42°C. Phenol-chloroform extracted and ethanol precipitated DNAs were fractionated on 6% Polyacrylamide-Urea Gels.

Northern blot analysis was carried out using the Northern-Max kit (Ambion) according to the manufacturer’s instructions. Five μg of total RNA were fractionated on 1% agarose-formaldehyde gel and transferred onto nylon membrane (Hybond+) through capillary blotting. Five pmol of radioactively-labelledlabelled primers (S1 Table) were used as probes. Hybridization and high-stringency washes were performed at 37°C, low-stringency washes at room temperature.

### Generation of plasmids and knockout strains

DNA manipulations were performed routinely as described for standard laboratory methods [54]. The MC58 Δ*hfq* mutant was described previously [55].

To generate sRNA null mutant strains, the upstream and downstream flanking regions of each sRNA were amplified by PCR using the primer pairs indicated in S1 Table. In a second round of PCR, the respective upstream and downstream flanking regions were fused through self-priming PCR, amplified with external primer pairs (S1 Table) and cloned as PCR products of about 1 kb carrying a BamHI or XmaI restriction site between upstream and downstream flanking regions in either pGEM-T (Promega) or pBluescript (pBS-KS, Novagen) vectors. These plasmids containing the sRNA flanking regions were digested with BamHI or XmaI and an erythromycin cassette [56] was inserted generating sRNA-KO::Ery^R^ plasmids (S1 Table). Following linearization these plasmids were used to transform MC58 and 2996 strains generating the sRNA knockout strains. The correct double homologous recombination event resulting on the knockout of the sRNAs was verified by PCR.

The same strategy was used to generate MC58 *deoR*, *gntR* and *hexR* null mutant strains, with the exception that each gene sequence was replaced with a kanamycin cassette [57]. Primers and plasmids used to generate the transcription factor knockout strains are listed in S1 Table.

### *In vivo* infant rat model

The infant rat model was used as described previously [58]. Briefly, bacteria were grown to log phase in GC medium, washed, and re-suspended at the desired concentration in PBS. Five- to 6-day-old pups from litters of outbred Wistar rats (Charles River) were challenged intra-peritoneally with 2996 wild-type and a isogenic 2996 sRNA knock-out mutant strain in a 1:1 ratio at the infective dose of 10^4^ CFUs, to establish mixed infections in the animals and determine the competitive index (CI). Eighteen hours after the bacterial challenge, blood samples were obtained by cheek puncture, and aliquots (100 μl of undiluted blood and 1:10 and 1:100 dilutions) were plated onto columbia agar and columbia agar + erythromycin or kanamycin, to select for viable cell counting of sRNA knocked-out bacteria. The numbers of CFU/ml of blood were determined after overnight incubation of the plates at 37°C in an atmosphere containing 5% CO_2_. Enumeration of wild type and mutant bacteria allowed for the determination of the CI ratio using the following formula: CI = (WT output/mutant output)/(WT input/mutant input). Statistical analysis was performed using Student’s t test.

### Ethics statement

All animal studies were carried out in compliance with current Italian legislation on the care and use of animals in experimentation (Legislative Decree 116/92) and with the Novartis Animal Welfare Policy and Standards. Protocols were approved by the Italian Ministry of Health (Authorization D.M. n. 166/2012—B) and by the local Novartis Animal Welfare Body (Research Project AWB 201202). Following infection, animals were clinically monitored daily for criteria related to their ability to feed, reactivity and motility, and cutaneous redness. After 18 hours all animals were alive and normally reactive, and were euthanized by cervical dislocation, as pre-established in agreement with Novartis Animal Welfare Policies.

## Results and Discussion

### Global analysis of *N*. *meningitidis* transcriptome in different conditions

In light of the multiple environments and stresses that meningococcus has to face during its pathogenesis [38], we set-up five *in vitro* growth conditions that likely mimic physiologically relevant signals for *N*. *meningitidis* infection such as iron starvation, oxidative stress, heat shock, carbon source limitation and glucose availability. The first three conditions involved brief environmental shocks of 5–12 min stress exposures (see Materials and Methods), and were used to enable identification of immediate gene expression responses to defined stresses. The second two conditions represented adaptive transcriptional responses of bacteria growing under different nutrient availability. In addition, we investigated differential transcription in the MC58 Hfq null mutant, due to the role of Hfq in reported meningococcus sRNAs regulatory circuits [30, 42, 55]. Finally, we analysed early stationary phase, which represents a multiple stress condition, as previous studies have reported a large number of sRNAs to be differentially expressed during this growth phase [59, 60].

**Fig 1:**
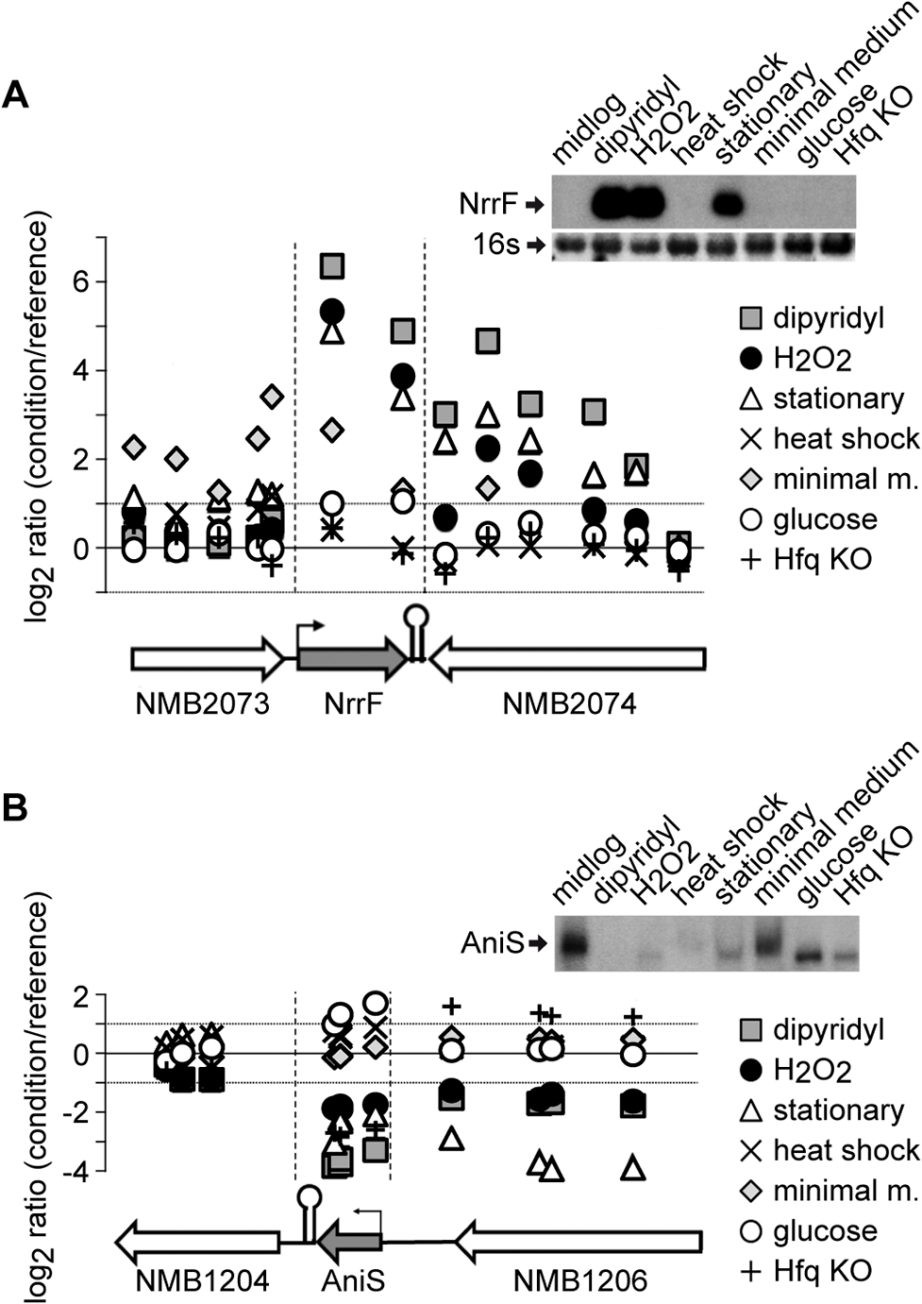
Validation of the high density data through NrrF and AniS expression profile. Expression profile of the NrrF (A) and AniS (B) sRNAs. Graphical representations of tilling array results during growth in the indicated conditions. The *y* axis shows the M value (log2ratio of expression of the experimental condition versus the reference), while on the *x* axis the genomic position of the probes is depicted in relationship to the schematic representation of the locus under the graph. ORFs and sRNAs are represented as white or grey horizontal arrows, respectively and the transcriptional starting sites and the terminators of sRNAs are indicated. Vertical black dashed lines indicate the position of sRNAs. Dotted black horizontal lines limit the background signal in which the probes are not considered to be regulated. Each dot in the graph corresponds to the average of the M values for one probe from three independent replicates in the indicated condition. On the upper right, experimental validation of the tilling array results by Northern blot (NrrF) or primer extension (AniS). 16s ribosomal RNAs provided a loading control for the Northern Blot experiment. The only notable inconsistency among microarray data and validation is the down-regulation of AniS in primer extension experiments during heat shock treatment. This discrepancy could be due to the fact that microarray probes and the primer used for validation do not recognize the same portion of AniS.

Total RNA was prepared as described in Materials and Methods and two-colour hybridizations on a previously described custom-made Agilent oligonucleotide tiling microarray [45] were performed to compare the transcriptional profiles of MC58 grown under the above stated conditions. Three independent two-colour microarray experiments were performed comparing pooled RNA from triplicate cultures of each condition, producing a total set of twenty-one experiments. Within the data set produced we first analysed the expression profile of the two known meningococcal sRNAs, NrrF [30] and AniS [42]. Fig 1A depicts a graph summarizing the microarray results for the expression of NrrF during all growth conditions tested. NrrF expression is induced at least 10-fold, when compared to the reference, during iron chelation (dipyridyl treatment), oxidative stress (H_2_O_2_ treatment) and stationary phase. These results are in agreement with previously reported NrrF regulation [30] and were also validated by Northern blot experiments (Fig 1A, top-right panel). Fig 1B shows analogous microarray results for AniS and the relative experimental validation, where it demonstrated over 2-fold repression during iron chelation, oxidative stress, stationary phase and in the Hfq mutant.

In addition to NrrF and AniS, six recently identified sRNAs induced in blood, named Bns (Blood-induced Neisserial Small RNAs) and validated by RACE experiments [45], appear differentially expressed in at least one out of the 21 experiments, when compared to the reference (S1A and S1B Fig). For three Bns (Bns1, Bns2 and Bns3) the regulation observed in one condition is maintained in all three replicates performed (S1A Fig). Recently [44], another sRNA has been identified in *N*. *meningitidis*, originally named σ^E^sRNA [44], which was subsequently identified to be the trans-activating CRISPR RNA (tracrRNA) of the type II CRISPR-Cas system in *N*. *meningitidis* [41]. Interestingly, in our analysis this sRNA is up-regulated during growth in minimal media and under heat-shock treatment (S1C Fig). This indicates that the synthesis of the tracrRNA of *N*. *meningitidis* responds to stress signals that may be relevant for infection.

In conclusion, we identified all previously reported sRNAs of *N*. *meningitidis*, as differentially expressed signals from probes mapping on non-coding intergenic regions.

### Dataset analysis and identification of novel putative small RNAs

In order to identify new regulated putative sRNAs, we submitted our microarray dataset through a recently developed bioinformatics tool that was used to define the changes in the whole transcriptional profile of *N*. *meningitidis* during growth in human blood, the chipSAD method [45], restricting the analysis to non-coding intergenic regions. By applying this procedure we obtained 592 intergenic signals that responded with at least 2-fold differential levels within at least one of the 7 different conditions and exhibited differential expression in all 3 replicates of that condition. Finally, by manual curation of the data using the criteria defined in Materials and Methods, we selected 98 intergenic regions as the most promising meningococcal sRNA candidates (S2 Table). Among the 91 putative sRNAs previously identified as being regulated in blood [45], 19 (20%) are reported in our final list (see the “Reference” column in S1 Table). However, if the analysis is performed without the exclusion of the probes that were regulated in only one single experiment (rather than being identified in all three replicates of each condition), we obtain a higher overlap with the list of sRNAs regulated in blood and thereby 71 (78%) of the previously identified intergenic regions are detected by our analyses as being differentially expressed intergenic signals.

**Fig 2:**
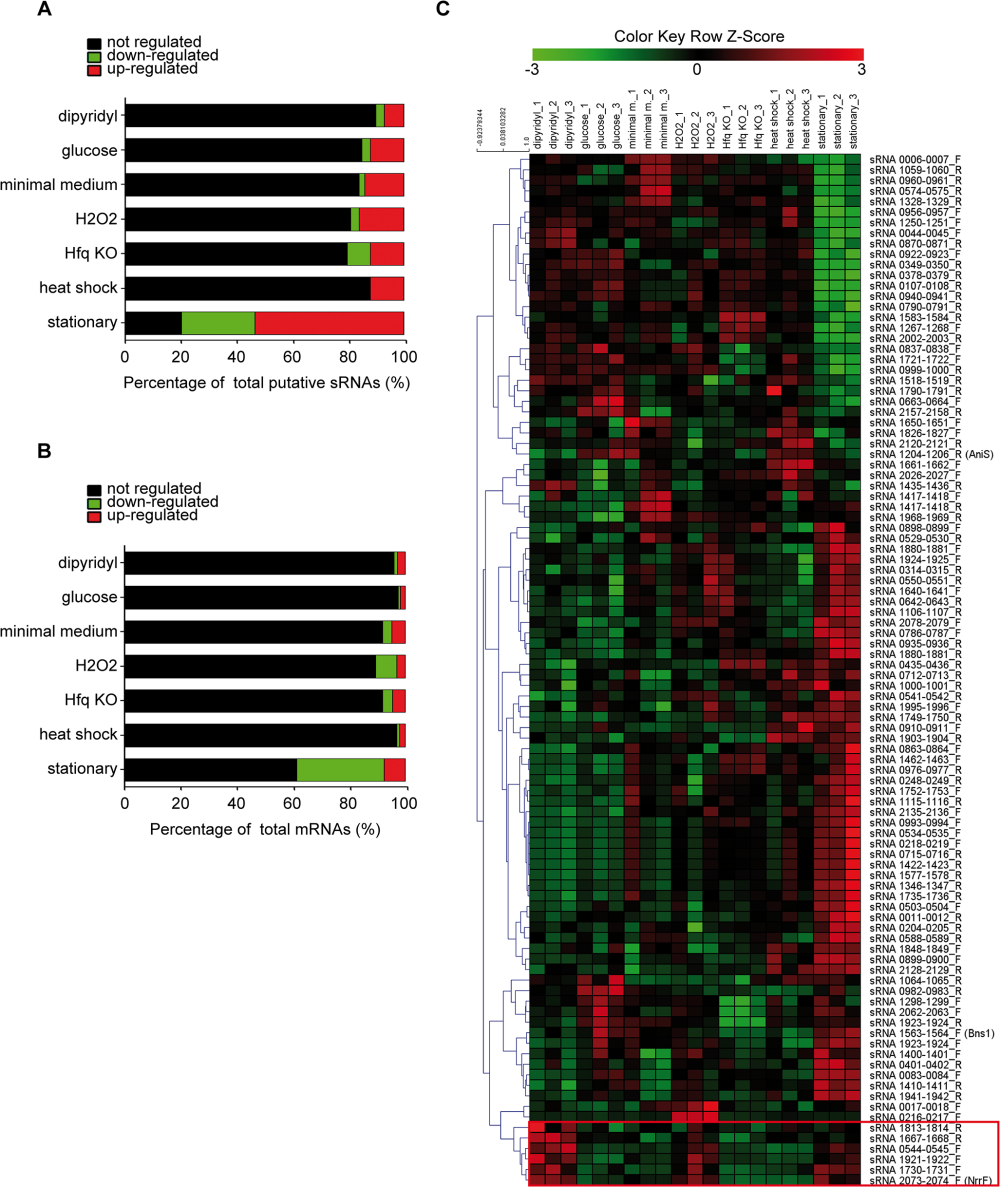
Identification of putative sRNA regulated in different conditions. (A-B) Bar plot percentages of the up- (red), down- (green) and not-regulated (black) total putative sRNAs (A) and mRNAs (B) in all the conditions tested. (C) Heatmap visualization of the 98 putative sRNAs according to their regulation in each individual microarray experiment (21 columns), showing an unsupervised hierarchical clustering analysis (complete linkage, Euclidean distance matrix) of putative sRNA, generated with the TM4 MeV v4.9 software. The colour key scale of raw-Z scores is defined on top of the heatmap. A red squared box underlines the group of sRNAs which cluster with NrrF.

Fig 2A shows the percentage of differentially expressed putative sRNAs identified in each of the different growth conditions and stresses that we analysed. The stationary phase of growth represents, as expected, the condition in which most differentially expressed intergenic transcripts were identified, comprising 68% and 76% of total up- and down-regulated putative sRNAs, respectively. In Fig 2B, the same analysis was performed on mRNAs. The majority of regulated mRNAs are modulated during stationary phase. Interestingly, we notice an anti-correlation in this condition: while the majority of modulated putative sRNAs are up-regulated, 80% of modulated mRNAs are down-regulated, suggesting that sRNAs in meningococcus may play an important role in the repression of gene expression during adaptation to stationary phase

Fig 2C shows an unsupervised heat-map with hierarchical clustering analysis of the 98 putative sRNAs throughout the 21 single experiments performed. Interestingly, the identifications of well-defined clusters of commonly modulated sRNAs, suggests the existence of sRNA-based networks in *N*. *meningitidis* involved in response to distinct environmental *stimuli*. The meningococcal Ferric Uptake Regulator (Fur) has been implicated in responding to both iron limitation and heat-shock stresses [61] and positively regulates the expression of succinate dehydrogenase by down-regulation of NrrF [30, 43]. However, previously reported data suggest that Fur can act via other unknown sRNAs and that therefore other unknown regulators for iron-responsiveness might be present in *N*. *meningitidis* [30, 61]. Here we identified a group of 5 sRNAs that cluster with NrrF, being highly induced in dipyridyl, heat shock and stationary phase (red square in Fig 2B and S2B Fig). Interestingly, sequence analyses identified Fur-box-like motifs proximal to the putative promoters of two sRNAs identified in this cluster, namely sRNAs 1730–1731_F and 1813–1814_R, while another sRNA, 1667–1668_R, is downstream of a gene coding for a hemoglobin receptor (S2A Fig), enforcing their putative roles as iron-responsive sRNA regulators possibly involved in iron homeostasis and adaptation of *N*. *meningitidis*. Accordingly, the genes that exhibit a common expression pattern across the 21 experiments and cluster together with these sRNAs comprise 60% of the Fur- and iron-regulated genes [61] of *N*. *meningitidis* (S2B and S2C Fig and S6 Table).

Altogether our analysis supports the hypothesis of a global network of sRNA transcription in *N*. *meningitidis*, possibly comprising at least a hundred sRNAs, differentially expressed under different growth conditions and likely during bacterial infection.

### Validation of novel sRNAs

**Fig 3:**
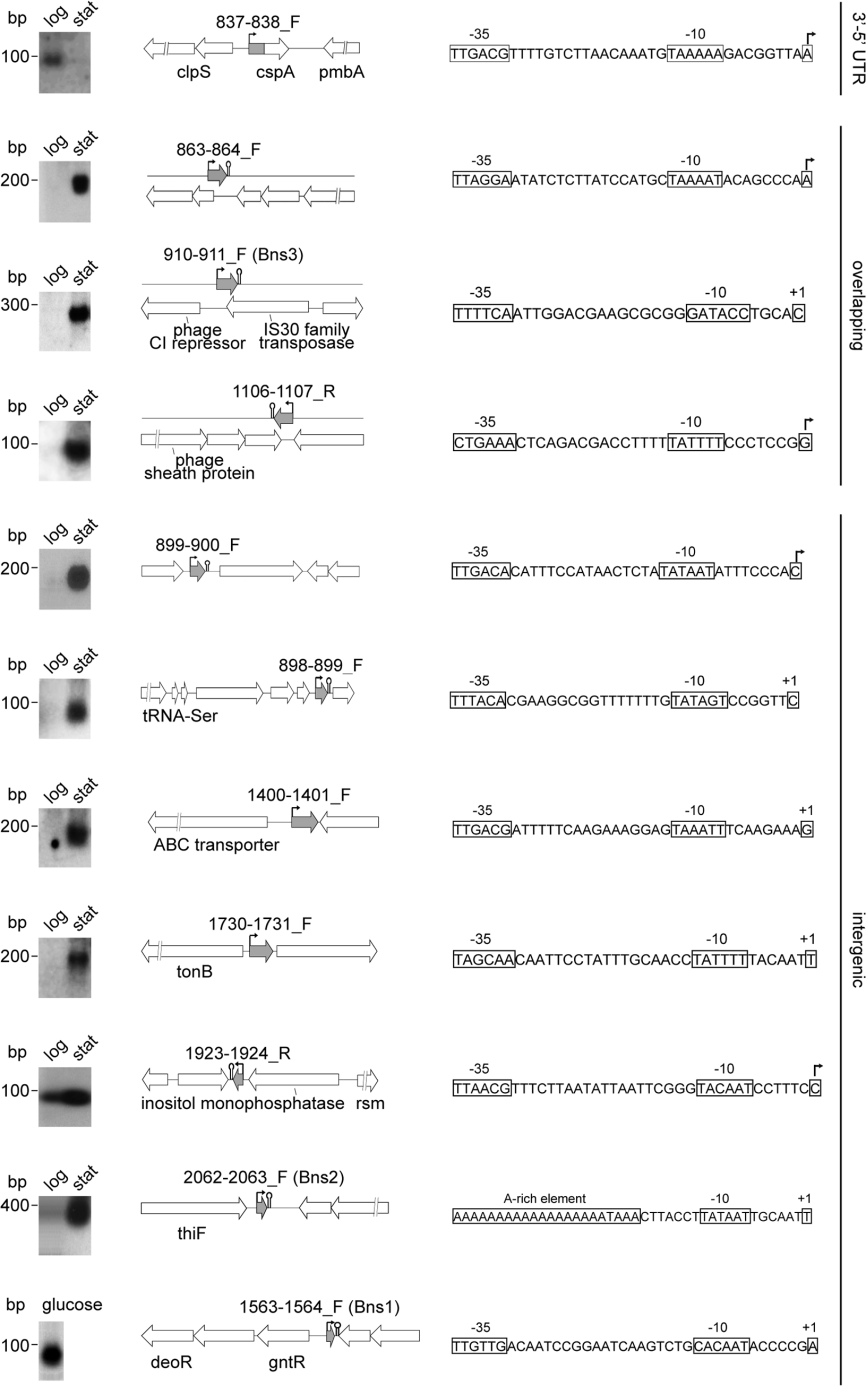
Validation and mapping of novel sRNAs. On the left panel, validation of 11 putative sRNAs by northern blots experiments performed with RNAs form logarithmic and stationary phase of growth, or RNA extracted in the condition of high expression of Bns1, according to microarray experiments. A molecular weight ladder is reported on the right. Uncropped version of these norther blots are provided in S4 Fig. Schematic representation of the locus (center) and promoter sequence (on the right) of each sRNA. White and grey arrows indicate ORFs and sRNAs, respectively. Transcriptional start sites and putative terminators are indicated by a bent arrow and a stem loop, respectively. Names of sRNAs and annotated genes are reported above and below the schematic representation of the locus, respectively. In the sequence, the +1 and the promoter elements -10 and -35 are indicated by boxes. Bent arrows indicate +1 sites that were determined by primer extension (related to S3 Fig). Predicted ORFs were identified for sRNAs 899–900_F, 910–911_F, 1106–1107_R and 1730–1731_F.

Out of the 98 candidates we selected 17 transcripts for validation by Northern blot, comprising: 3 previously validated Bns (Bns1/1563-1564_F, Bns2/2062-2063_F and Bns3/910-911_F); 4 previously identified putative transcripts in *ex vivo* human blood (IG24/898-899_F, IG25/899-900_F, IG48/1417-1418_R, and IG73/1923-1924); and 10 new putative sRNAs. Radiolabelled oligonucleotide probes hybridized to total RNA extracted from both logarithmic and stationary growth phases resulted in 10 signals, corresponding to small transcripts in the range of 100–300 nt in length, in either one or both conditions (Fig 3, left panel). As expected from the microarray data, the signal from 837–838_F was detected only in RNA extracted from logarithmic phase, while the other signals from 863–864_F, 898–899_F, 899–900_F, 910–911_F, 1106–1107_R, 1400–1401_F, 1730–1731_F, 1923–1924_R, 2062–2063_F were significantly induced in stationary phase (Fig 3A). An additional signal was detected for Bns1 only in Northern blot performed on total RNA from C6 minimal medium supplemented with glucose (Fig 3, lower left panel), a condition in which it appears to be induced in the microarray results. Therefore, 11 out of 17 candidates analysed were confirmed, including 3 Bns sRNAs previously validated by RACE (Bns1/1563-1564_F, Bns2/2062-2063_F, Bns3/910-911_F) and 3 previously identified as putative transcripts (IG24/898-899_F, IG25/899-900_F and IG73/1923-1924) [45]. We detected no signals for 6 of the candidates, (namely, 216–217_F, 534–535_F, 544–545_F, 1813–1814_R, 1921–1922_F and the previously identified IG48/1417-1418_R) even under the conditions in which the microarray results indicated induction. This may suggest either that Northern blot experimentation exhibits limited sensitivity when compared to the microarray hybridization and detection protocol, or these putative sRNAs may represent false positive results.

Sequence analysis of validated sRNAs identified putative promoters and rho-independent terminators of sRNAs (Fig 3, central and right panels). We selected five new sRNAs and performed primer extension experiments to confirm their transcriptional starting sites (+1) and the relative promoter sequences (Fig 3, right panel and S3 Fig). Among these, we distinguished different classes of transcripts according to their position relative to annotated ORFs (Fig 3, central panel): intergenic sRNAs, sRNAs partially overlapping an ORF in the opposite strand, and 3’- or 5’-UTR processed sRNAs.

Two intergenic probes upstream of NMB0838 gave signals of differential expression in the microarray results, but similar differentially regulated signals were also detected by contiguous probes mapping on NMB0838 (data not shown). Northern blot experiments (Fig 3, left panel) detected a specific intergenic transcript of about 100 nt, the sRNA 837–838_F, possibly representing the result of a processing of the 5’UTR region of a primary longer transcript from NMB0838. The sRNA 863–864_F is a transcript of about 240 nt with a putative terminator at its 3’ end (Fig 3, second row) and overlaps 28 nucleotides of NMB0863 in the antisense strand, representing an example of gene-overlapping sRNA. Conversely, the sRNA 899–900_F is an example of intergenic sRNA, having its promoter, +1 and putative terminator sequences in the intergenic region between the two flanking ORFs (Fig 3, fifth row). The 98 putative sRNAs listed in S2 Table were classified according to their position relative to annotated ORFs, as being part of one of these 3 classes: 3’-5’UTR processed sRNAs, overlapping sRNAs and intergenic sRNAs. The presence of different classes of sRNAs in the meningococcal genome is in accordance with the great heterogeneity of these molecules [1, 2]. In particular, as *N*. *meningitidis* has to face specific ecological niches [62], it is tempting to speculate that only a restricted number of sRNAs have gene expression regulatory functions. Many sRNAs in enterobacteria are divergently transcribed and encoded next to their own regulators (e.g. SgrS-SgrR, OxyS-OxyR, GcvB-GcvA) [63–65]. Interestingly, we also identified putative sRNAs (namely 910–911_F, 204–205_R, 1650–1651_F, 1995–1996_F, 0216–0217_F and 712–713_R) within intergenic regions flanking the genes encoding the LexA, Fur, Lrp and GlnB transcriptional regulators, as well as the alternative sigma factors of meningococcus, sigma32 and sigma54, respectively. Future experiments might be addressed to characterizing one or more of these regulatory sRNA candidates for their functional interactions with their neighbouring regulators.

Putative ORFs have been identified within the sequence of validated sRNAs 899–900_F, 910–911_F, 1106–1107_R and 1730–1731_F, raising the question whether these transcripts may code for small proteins or peptides [66]. However, no ribosomal binding sites could be identified upstream of the start codons for these ORFs, and no function could be inferred from the predicted amino acid sequences (data not shown). Other sRNAs may have structural roles [67], act post-translationally on proteins [6], or have dedicated roles in targeting of DNA sequences, like those involved in the CRISPR/Cas bacterial defence system [41]. Additionally, a newly discovered sRNA in *N*. *gonorrhoea* has recently been proposed to facilitate the formation of a G-quadruplex DNA structure involved in antigenic variation the gonococcal pilus [40].

The sequences of the 11 validated sRNAs were found to be highly conserved and associated to the same loci across *Neisseria spp*. genomes (S3 Table). Notably, 5 sRNAs were found to be absent in the non-pathogenic *N*. *lactamica* (namely, IG24/898-899_F, IG25/899-900_F, Bns3/910-911_F, 1106–1107_R and 1730–1731_F), suggesting a possible role in pathogenic-specific processes. In addition 3 sRNAs, IG24/898-899_F, Bns3/910-911_F and 1400_1401_F, are associated to transposable elements, suggesting a recent acquisition of these functions via horizontal gene transfer (S3 Table).

Altogether our experimental validation by Northern blot and primer extension verified and confirmed the presence of 8 new and 3 previously identified sRNAs, respectively, which are also highly conserved across meningococcal genomes. Moreover, we identified three different classes of sRNAs according to their genomic position, for which we have described three representative case studies.

### Bns1 is involved in regulating metabolic processes in a circuit with GntR

**Fig 4:**
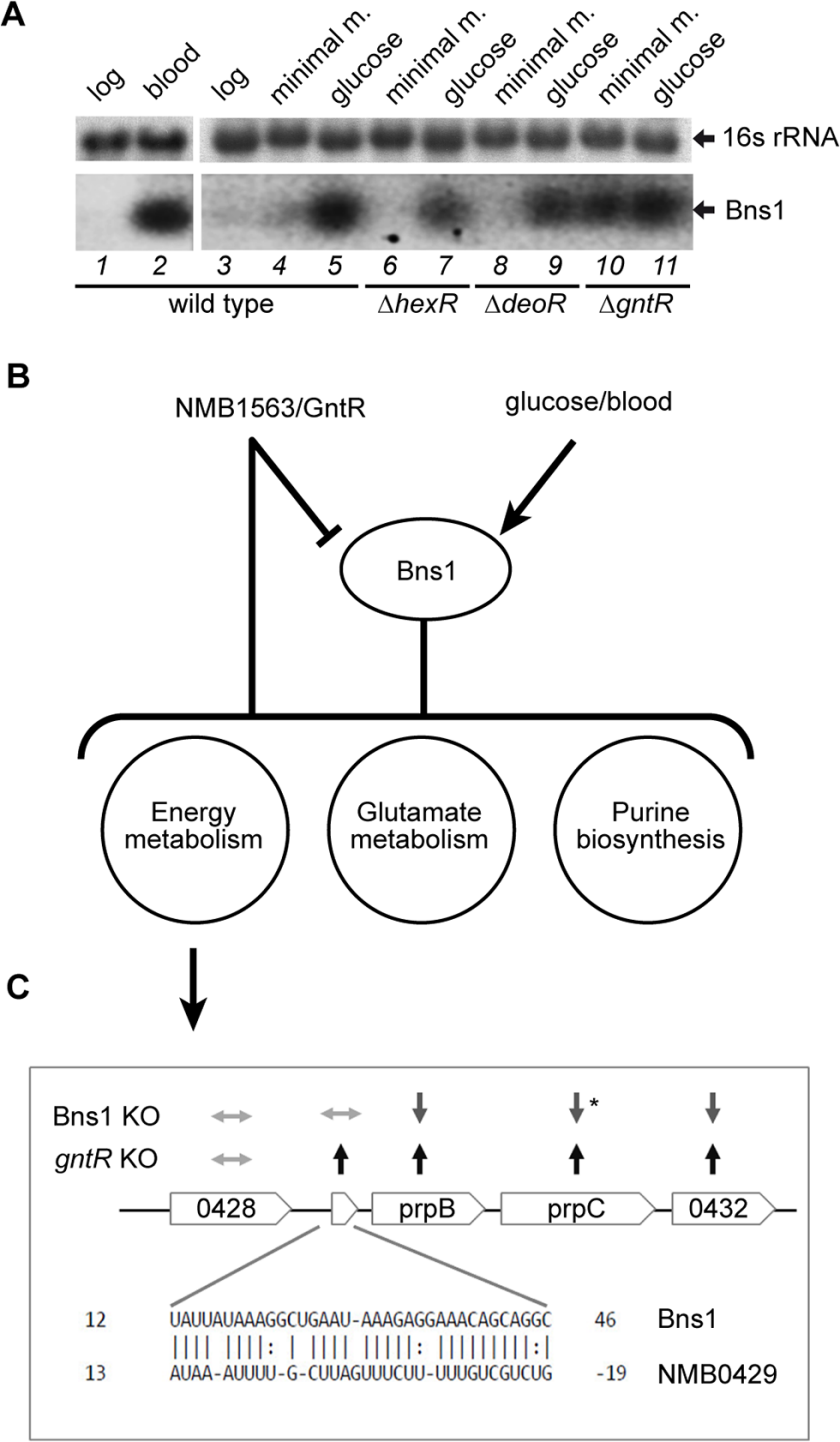
Bns1 regulatory network. (A) Northern blot experiments showing regulation of Bns in blood (left panel) and in minimal media (MM) or minimal media with 1%(w/v) glucose (gluc) in either wild type or indicated mutant strains (right panel). 16s rRNA is provided as a loading control. (B) Proposed model of Bns1 regulatory network (See text for details). (C) Bns1 target prediction on NMB429 and regulation of the operon NMB429-NMB432 in Bns1 and *gntR* KO strains. Asterisk indicates a p-value = 0.052 for the down-regulation of *prpC* in Bns1 KO.

One of the validated sRNAs, Bns1, was differentially induced by glucose (1.85-fold induction, S2 Table) and was previously identified as a sRNA induced under *ex vivo* exposure to human blood [45]. By Northern blot we confirmed its regulation: while this sRNA is not synthesized under standard *in vitro* conditions, the addition of glucose (55 mM in the minimal media) or exposure to the glucose-rich human blood (4.5 mM [68]) results in its induction (Fig 4A, lanes 1–5).

Bns1 is divergently transcribed from an operon that includes orthologues of the DeoR and GntR families of transcriptional regulators, NMB1561 and NMB1563, respectively (Fig 3, central panel in last row). Importantly, the association of Bns1 with these genes is conserved among different Neisserial genomes (S3 Table). These proteins have been reported to act as repressors in carbon metabolism of different bacteria [69–79]. Another key regulator of the central carbon metabolism is the HexR regulator [80]. To investigate whether one of these is involved in regulation of Bns1, we generated knock-out (KO) mutants in MC58 strain of NMB1563 and NMB1561 and also NMB1389, coding for an orthologue of HexR. Total RNAs were extracted from these KO strains grown in either minimal media or minimal media supplemented with 1% (w/v) glucose and assayed by Northern blot experiments. Fig 4A (lanes 4–9) shows that while in the *deoR* and *hexR* KO the sRNA is regulated as in the wild type, in the *gntR* KO background, Bns1 is synthesized also in minimal media without glucose, suggesting a negative regulation of GntR on the sRNA in the absence of carbon sources.

We then performed microarray experiments of wild type versus Bns1 or *gntR* KO mutants (S4 and S5 Tables, respectively), to identify the genes which they regulate. We profiled global gene expression of Bns1 KO against the wild type under minimal media and minimal media+glucose growth conditions, while the GntR KO was assessed after growth in GC medium where the nmb1563 *gntR* gene is well expressed. Results show that 46 genes were differentially regulated by Bns1 (28 up-regulated and 18 down-regulated, pval ≤ 0.05; S4 Table) and 141 genes were differentially regulated in the knock out mutant of the transcriptional regulator GntR (110 up-regulated and 31 down-regulated, pval ≤ 0.05; S5 Table). Interestingly, almost one third (32.6%) of Bns1 regulated genes are also regulated by the deletion of *gntR*, suggesting that the sRNA and the transcriptional regulator share, at least in part, the same regulatory network. Bns1 regulates genes involved in energy metabolic processes (NMB0435/acetate kinase, NMB0604/ alcohol dehydrogenase, NMB0954/citrate synthase, NMB1493/carbon starvation protein A, NMB0430/ carboxyphosphonoenolpyruvate phosphonomutase, NMB0567/Na(+)-translocating NADH-quinone reductase, subunit C) and transporters of metabolic substrates (NMB1362/oxalate/formate antiporter, NMB0543/L-lactate permease). In addition, both glutamate metabolism (NMB1710/ glutamate dehydrogenase, NMB1074/acetylglutamate kinase) and purine biosynthesis (NMB0690/ amido-phosphoribosyl-transferase, NMB0983/phosphoribosyl-aminoimidazole-carboxamideformyl transferase) are altered. Moreover, several genes found to be highly regulated by Bns1, are also found to be up-regulated in blood as well as under *in vitro* conditions where glucose is the only carbon source, such as NMB1362 (oxalate/formate antiporter), NMB0378 (putative phosphate permease), NMB0663 (*nspA*), NMB0615 (putative ammonium transporter *amtB*) and NMB1710 (glutamate dehydrogenase *gdhA*), therefore depicting glucose as an important signal of the *in vivo* environment to which this sRNA responds.

Among the genes that are regulated in both the Bns1 and GntR knockout, one third is inversely regulated, while the others are regulated in the same way. Although we do not provide evidence of direct regulation of these targets by the respective regulators, TargetRNA predictions for Bns1, as previously reported [45], retrieves NMB0429 as the best hit (Fig 4C). NMB0429 is part of an operon, strongly up-regulated in blood [45], together with NMB0430/*prpB* (2-methylisocitrate lyase) and NMB0431/*prpC* (methylcitrate synthase) that is implicated in propionate metabolism. Interestingly, the entire operon is up-regulated in the *gntR* (Fig 4C and S5 Table) and down-regulated in Bns1 mutant, respectively (Fig 4C and S4 Table). Altogether, these data indicate that Bns1 is repressed by GntR in the absence of carbon sources and that Bns1 acts as a positive regulator of NMB0429-prpB-prpC operon, possibly by stabilizing mRNAs. De-repression of Bns1 in the *gntR* mutant background, or Bns1 up-regulation in response to carbon source availability (e.g. glucose or human blood [45]), leads to up-regulation of genes in the NMB0429-prpB-prpC operon. We therefore propose a model (Fig 4B and 4C), in which Bns1 and the carbon metabolism regulator GntR share the same regulatory network, controlling key processes important for adapting *N*. *meningitidis* to the host environment. Importantly, *prpC* was recently shown to be up-regulated in chemically defined media with glucose and to be important for utilization of propionic acid as an alternative carbon source [81]. Finally, the observation that Bns1 is induced in blood correlates well with the concentration of glucose in this niche of infection, which is higher than in other host reservoirs such as saliva or cerebrospinal fluid [68], and supports a role in meningococcal pathogenesis.

### sRNAs involved in *N*. *meningitidis* survival *in vivo*

In order to establish the role of validated sRNAs on meningococcal fitness *in vivo*, we generated knockout mutants in *N*. *meningitidis* strain 2996 and performed a competitive index (CI) assay in the infant rat model of bacteremia.

**Fig 5:**
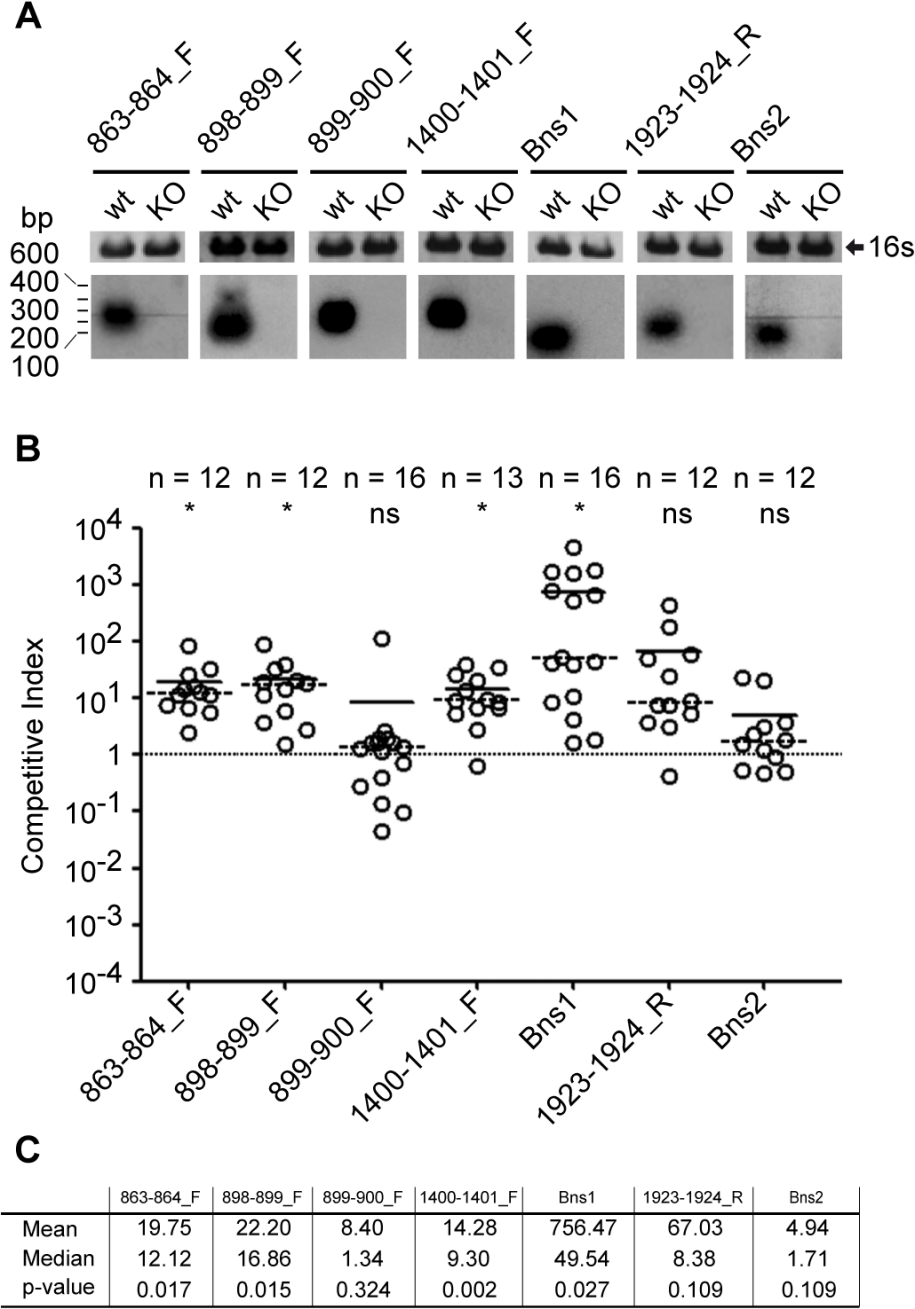
Effects of sRNA deletions on meningococcal infection. (A) Northern blots experiments validating the deletion of the indicated sRNAs. RNA was extracted from 2996 wild type (WT) and relative deletion strains grown until stationary phase for all sRNAs, except for Bns1/1563-1564_F for which the growth condition was minimal medium supplemented with glucose. 16s rRNA is provided as a loading control. (B) Competitive index (CI) from infant rats infected with WT *N*. *meningitidis* 2996 and the indicated sRNA KO strains at a 1:1 ratio. Solid circles indicate individual animals. Horizontal solid and dashed black lines indicate means and medians for each group, respectively. A horizontal dotted line is set to CI = 1, indicating no effect on meningococcal infection due to deletion of the respective sRNAs. The number of animals in each group and the results of statistical analysis are shown above the graph (2-tails, unpaired T-test analysis were performed to assess the statistical significance for each group of CIs to be different from 1; * = pval ≤ 0.05, ns = no statistical significance). (C) Table summarizing result of CI index assay with statistical analysis.

We successfully deleted seven validated sRNAs, comprising two Bns molecules (Bns1 and Bns2), as demonstrated in Northern blots in Fig 5A. Growth curves in GC rich medium showed no significant differences for these KO mutants when compared with wild type strain (data not shown). Infant rats were infected with the infective dose of 10^4^ CFUs of 2996 wild type and individual sRNAs knockout (KO) strains at a ratio 1:1 by intra-peritoneal injection. Bacterial survival of wild type and sRNA KO strains in the blood was determined by CFUs counting on selective and non-selective agar media. Fig 5B shows the CI of each infant rat for each sRNA KO. In four out of seven sRNAs (Bns1, 863–864_F, 898–899_F and 1400–1401_F) a statistically significant CI greater than one was observed, indicating that more wild type bacteria survived in infant rat blood than mutant strain bacteria. These observations therefore showed that knocking out these sRNAs affects *in vivo* survival of *N*. *meningitidis* in the bacteremia model. The remaining three sRNA (Bns2, 899–900_F, 1923–1934_R) KO strains exhibit CI not significantly different to one, demonstrating that the lack of their expression is not sufficient to alter meningococcal fitness in the *in vivo* infant rat model of bacteremia. Nonetheless, we cannot exclude that some of them, such as 899–900_F, which is not conserved in non-pathogenic *Neisseria* (S3 Table), may be involved in functions related to meningococcal pathogenesis that are not assessed by the model used.

Interestingly, the knockout of Bns1 generated the greatest CI values (mean = 756.47, median = 49.54, as reported in Fig 5C), identifying a relevant role on meningococcal survival during infection. In a genome-wide screening of 2850 insertional mutants of *N*. *meningitidis*, Sun and colleagues [82] identified 73 genes that are essential for bacteremia, comprising genes involved in the same processes in which Bns1 has been implicated through the microarray experiments: energy metabolism and transport of metabolic molecules, amino acid biosynthesis and purine, pyrimidine, nucleosides and nucleotides biosynthesis. In particular, four genes (NMB1362/oxalate/formate antiporter, NMB0792/NadC transporter family, NMB0543/L-lactate permease, NMB1710/ glutamate dehydrogenase) identified by Sun and colleagues [82], are deregulated in the Bns1 KO. The role of the sRNA in regulation of energy metabolism and the attenuated phenotype of its KO mutant, suggest a tight correlation between the carbon metabolism of this organism and its survival in the host environment during bacteremia. The response to carbon source availability is of special importance for bacteria, which can use several mechanisms to adapt to changes in carbohydrate composition. These mechanisms can include induction of a specific carbohydrate transport and the utilization system by the presence of the corresponding carbon source or alternatively their repression when a more efficiently utilizable carbohydrate is present or carbon catabolite repression (CCR). As we report here for Bns1, several studies describe the emergence of sRNAs as additional regulators of metabolism with several found to act at the interface of bacterial metabolism and virulence factor expression. For instance in *E*.*coli*, Spot 42, an Hfq-binding sRNA, selectively turns off the synthesis of GalK that is required for galactose metabolism when a preferred carbon source such as glucose is available [83] and synergizes with the global transcriptional regulator cyclic AMP (cAMP) receptor protein (CRP) to contribute to the overall efficiency of CCR [84]. In the Nm genome, an orthologue of CRP is not evident suggesting that other mechanisms of regulation of metabolism may exist such as the GntR/Bns1 regulatory circuit. Here we suggest that the GntR/Bns1 regulatory circuit could be involved in regulating the propionate catabolism, in response to the availability of different carbon sources. Interestingly in *E*. *coli* it has been shown that the propionate catabolic (*prpBCDE*) genes are regulated by the global regulator of sugar transport, the cAMP-CRP complex, and inhibition of the *prpBCDE* promoter is encountered during rapid sugar uptake and metabolism [85].

In conclusion, we report for the first time the discovery of meningococcal sRNAs that affect bacteremia. Moreover, we have identified the Bns1 sRNA as an important regulator of *N*. *meningitidis* bacterial metabolism and have shown that it plays a crucial role for Nm survival in the infant rat model. Finally, the data presented here provide a significant resource for future studies required to shed light on sRNA-mediated regulation of key virulence determinants of *N*. *meningitidis*.

## Supporting Information

S1 Fig Expression profile of previously described meningococcal sRNAs within the array dataset. Graphical representations of tilling array results concerning six sRNAs regulated in blood [45] (A and B) and the σ^E^sRNA homologue in MC58 [44] (C). The conditions in which the sRNAs were differentially expressed are indicated as grey squares or black circles. Each dot represents either the average of the M values for one probe from three independent replicates in one condition (A and C), or the M value in a single experiment (B). The *y* and *x* axis show the M value (log2ratio of expression of the experimental condition versus the reference), and the genomic position of the probes, respectively. A schematic representation of the locus is shown under each graph. White and grey arrows indicate ORFs and sRNAs, respectively. Vertical black dashed and horizontal black dotted lines limit the sRNAs and the background signal in which the probes are not considered to be regulated, respectively.
(TIFF)

S2 Fig Analysis of novel sRNAs clustering with NrrF. (A) Schematic representation of the locus (on the left) and sequence (on the right) of the 5 novel sRNAs clustering with NrrF. White and grey arrows indicate ORFs and sRNAs, respectively. Transcriptional start sites are indicated by a bent arrow. In the sequence, putative promoter elements are boxed, and probes giving a differentially regulated signal in the dipyridyl microarray experiments are highlighted in grey. sRNAs 1730–1731_F and 1813–1814_R display Fur-box-like motifs near putative promoters (highlighted in bold in the sequence). Motif search was performed using the fuzznuc algorithm, allowing up to 8 mismatches from the meningococcal Fur-box consensus. (B) Expression profile of mRNAs and sRNAs which cluster with NrrF, along the 21 experiments. (C) Overlap between NrrF cluster genes and Fur/iron regulated genes (see also S6 Table).
(TIFF)

S3 Fig Primer extension on validated sRNAs. Primer extension experiments on five validated sRNAs, showing the identified +1. Growth culture conditions in which RNA were extracted are indicated on top of each panel. A G+A molecular weight ladder is shown on the left.
(TIFF)

S4 Fig Uncropped version of northern blots from Fig 3. sRNAs are indicated by arrows, while asterisks indicate signals from transcripts at the same size range as 23s and 16s rRNAs. 16s rRNA northern blots are provided as loading controls.
(TIFF)

S1 Table Primers, plasmids and strains used in this study. a = restriction sites are indicated in the sequences in lowercase and underlined. b = NB stands for Northern Blot, PE stands for Primer Extension, KO stands for Knock Out generation.
(XLSX)

S2 Table Identified putative sRNAs. * = sRNAs experimentally validated through northern blot.- = putative sRNAs submitted to northern blot validation, without having positive results. a = F and R mean forward and reverse strand, respectively. b = > and < mean forward and reverse strands, respectively. c = the different conditions/stresses analyzed are indicated as follow: dipyridyl (DIP), oxidative stress (H2O2), heat shock (HS), stationary phase of growth (STAT), growth in minimal media (MM), growth with glucose as the only carbon source (GLUC), RNA from the Hfq null mutant (HFQ). For each putative sRNA only one M value (log2 Cy5/Cy3) from the microarray dataset is reported for each condition/stress, representing the average of M values from all the probes mapping on the sRNA sequence in all the 3 replicates performed. d = genomic coordinate of the first nucleotide of the first probe mapping on the relative putative sRNA sequence, giving a signal of differential regulation in the microarray dataset. e = genomic coordinate of the last nucleotide of the last probe mapping on the relative putative sRNA sequence, giving a signal of differential regulation in the microarray dataset. f = Y and N indicate the presence or absence of putative rho-independent terminators, respectively. g = intergenic indicates a putative sRNA located in an intergenic region between two annotated ORFs; overlapping indicates a putative sRNA partially overlapping an annotated up or down ORF in the opposite strand; 3'-5' UTR indicates a putative sRNA that is located in a possible untranslated region of an annotated ORF. h = previously described putative sRNA are indicated. All IGs refers to Del Tordello et al., J.Bact. 2012. i = AniS sRNA is annotated as NMB1205.
(XLSX)

S3 Table Analysis of sequence conservation of validated small RNAs. a = percentage of the Nm MC58 sRNA sequence retrieved by BLASTN 2.2.29 search of neisserial genomes. b = + means that BLASTN hit locates sRNAs within the same locus as in Nm MC58.
(XLSX)

S4 Table Genes regulated in sRNA NMB1563-1564_F knock out. * = commonly differentially expressed genes by sRNA NMB1563-1564 KO in both minimal media and glucose. a = comparison of global gene expression profile of MC58 wild type and MC58 sRNA1563-1564 KO strains grown until midlog phase in minimal media (C6). b = comparison of global gene expression profile of MC58 wild type and MC58 sRNA1563-1564 KO strains grown until midlog phase in minimal media supplemented with 1% glucose. c = up- and down-regulated genes were selected setting log2ratio cut-off ≥ 1 and ≤ -1, respectively. d = p-value cut-off was set ≤ 0.05.
(XLSX)

S5 Table Genes regulated in NMB1563/GntR knock out. * = commonly differentially expressed genes by sRNA NMB1563-1564 KO and GntR KO. a = up- and down-regulated genes were selected setting log2ratio cut-off ≥ 1 and ≤ -1, respectively. b = p-value cut-off was set ≤ 0.05.
(XLSX)

S6 Table Overlap between Fur/iron regulon and NrrF cluster. a = from Delany I. et al., J.Bact., 2006.
(XLSX)
