# Supplementary figures and images for: Global Transcriptome Analysis Reveals Small RNAs Affecting Neisseria meningitidis Bacteremia
Source: PLoS One. 2015 May 7;10(5):e0126325. doi: 10.1371/journal.pone.0126325 (PMC4423775; doi:10.1371/journal.pone.0126325)

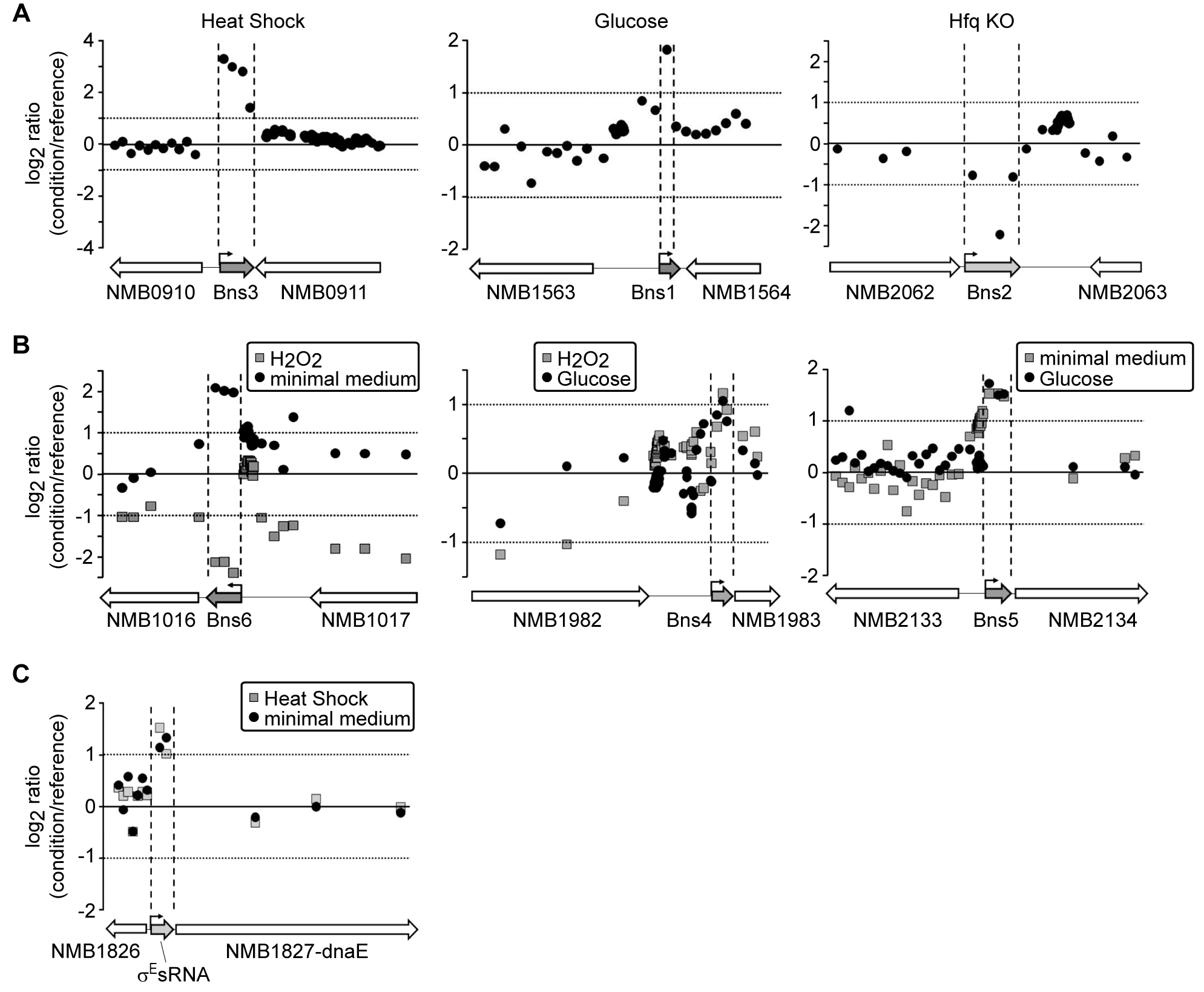

Supplement: S1 Fig — Graphical representations of tilling array results concerning six sRNAs regulated in blood [45] (A and B) and the σEsRNA homologue in MC58 [44] (C). The conditions in which the sRNAs were differentially expressed are indicated as grey squares or black circles. Each dot represents either the average of the M values for one probe from three independent replicates in one condition (A and C), or the M value in a single experiment (B). The y and x axis show the M value (log2ratio of expression of the experimental condition versus the reference), and the genomic position of the probes, respectively. A schematic representation of the locus is shown under each graph. White and grey arrows indicate ORFs and sRNAs, respectively. Vertical black dashed and horizontal black dotted lines limit the sRNAs and the background signal in which the probes are not considered to be regulated, respectively. (TIFF) [file pone.0126325.s001.tiff]

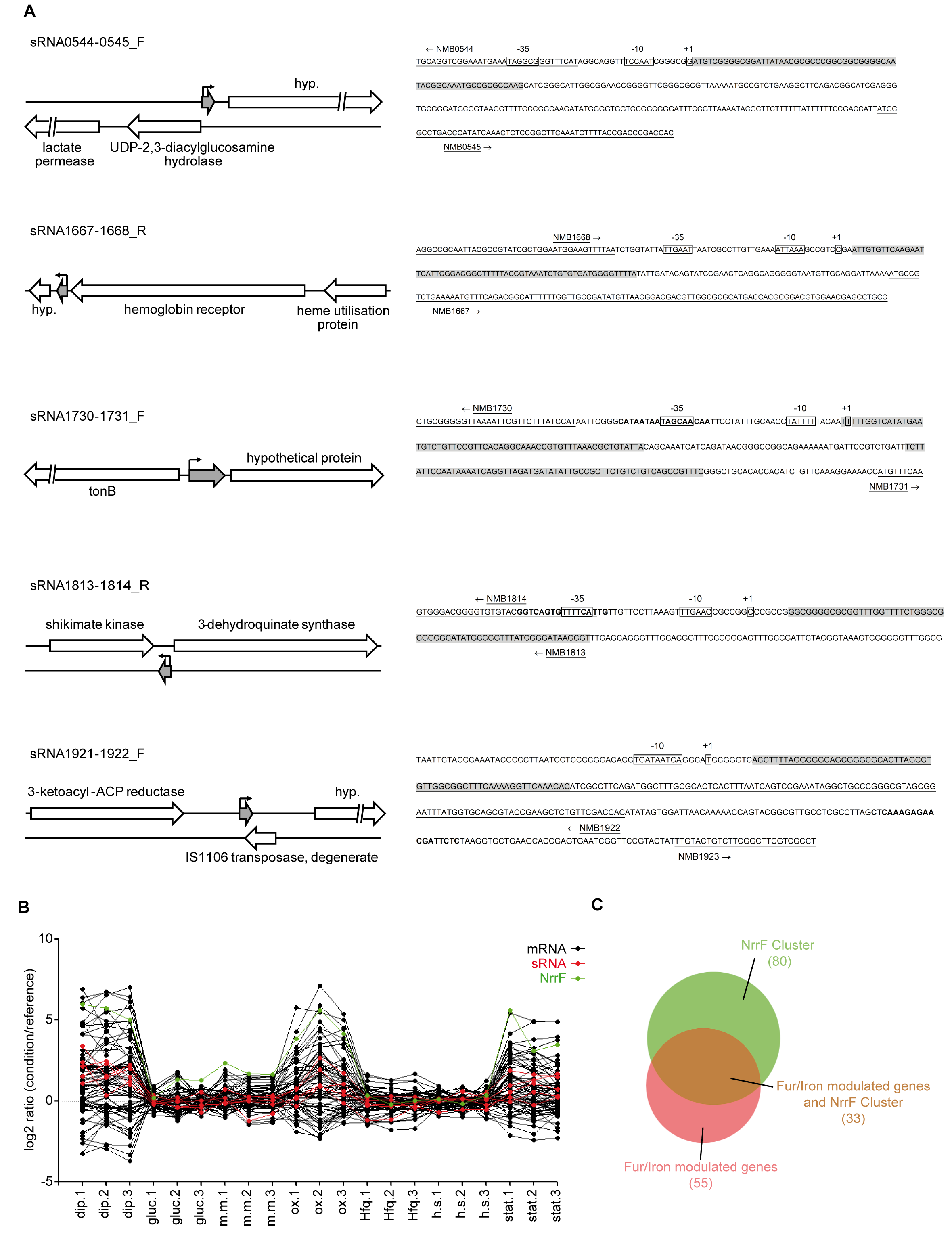

Supplement: S2 Fig — (A) Schematic representation of the locus (on the left) and sequence (on the right) of the 5 novel sRNAs clustering with NrrF. White and grey arrows indicate ORFs and sRNAs, respectively. Transcriptional start sites are indicated by a bent arrow. In the sequence, putative promoter elements are boxed, and probes giving a differentially regulated signal in the dipyridyl microarray experiments are highlighted in grey. sRNAs 1730–1731_F and 1813–1814_R display Fur-box-like motifs near putative promoters (highlighted in bold in the sequence). Motif search was performed using the fuzznuc algorithm, allowing up to 8 mismatches from the meningococcal Fur-box consensus. (B) Expression profile of mRNAs and sRNAs which cluster with NrrF, along the 21 experiments. (C) Overlap between NrrF cluster genes and Fur/iron regulated genes (see also S6 Table). (TIFF) [file pone.0126325.s002.tiff]

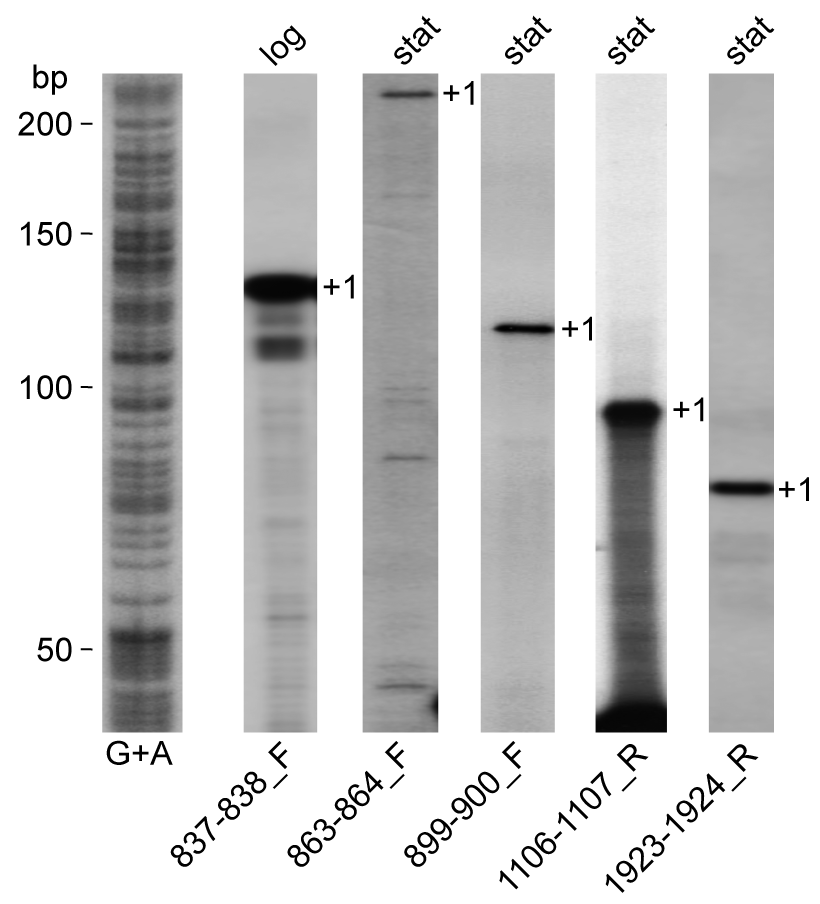

Supplement: S3 Fig — Primer extension experiments on five validated sRNAs, showing the identified +1. Growth culture conditions in which RNA were extracted are indicated on top of each panel. A G+A molecular weight ladder is shown on the left. (TIFF) [file pone.0126325.s003.tiff]

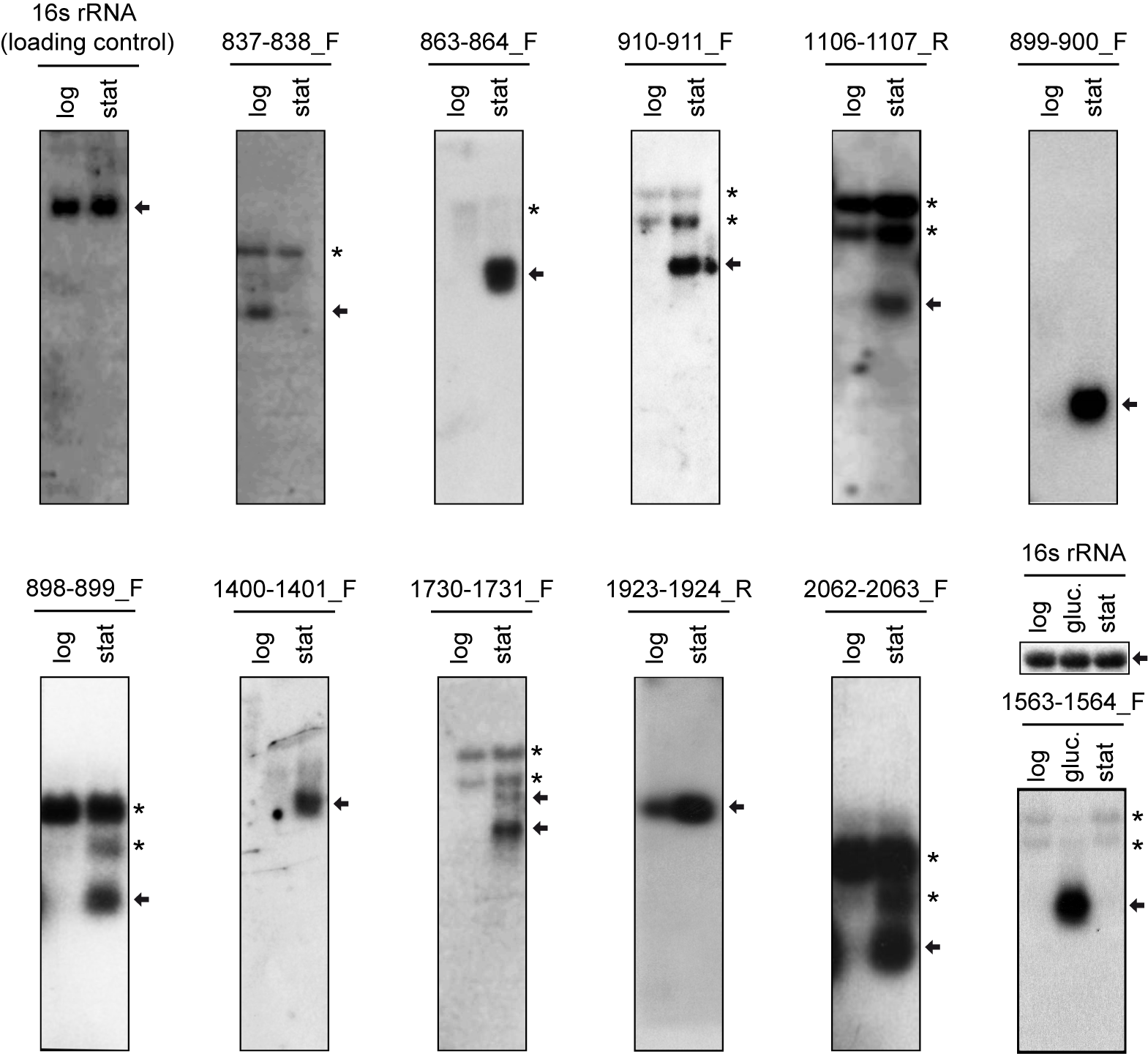

Supplement: S4 Fig — sRNAs are indicated by arrows, while asterisks indicate signals from transcripts at the same size range as 23s and 16s rRNAs. 16s rRNA northern blots are provided as loading controls. (TIFF) [file pone.0126325.s004.tiff]
